# Treatment-seeking and recovery among young undernourished children post-hospital discharge in Bangladesh: A qualitative study

**DOI:** 10.1371/journal.pone.0274996

**Published:** 2022-09-23

**Authors:** Md. Fakhar Uddin, Sassy Molyneux, Kui Muraya, Julie Jemutai, James A. Berkley, Judd L. Walson, Md. Alamgir Hossain, Md. Aminul Islam, Scholastica M. Zakayo, Rita Wanjuki Njeru, Tahmeed Ahmed, Mohammod Jobayer Chisti, Haribondhu Sarma

**Affiliations:** 1 Nutrition and Clinical Services Division, International Centre for Diarrhoeal Disease Research, Bangladesh (icddr,b), Dhaka, Bangladesh; 2 KEMRI-Wellcome Trust Research Programme, Kilifi, Kenya; 3 Centre for Tropical Medicine and Global Health, University of Oxford, Oxford, United Kingdom; 4 KEMRI-Wellcome Trust Research Programme, Nairobi, Kenya; 5 Department of Global Health, Medicine, Pediatrics and Epidemiology, University of Washington, Seattle, WA, United States of America; 6 National Centre for Epidemiology and Population Health, The Australian National University, Canberra, Australia; PLOS (Public Library of Science), UNITED KINGDOM

## Abstract

**Introduction:**

Post-hospital discharge mortality is high among undernourished children in many low and middle-income countries. Although a number of quantitative studies have highlighted a range of potential socio-cultural, economic and health system factors influencing paediatric post-discharge treatment-seeking and recovery, few studies have explored family and provider perspectives of the post-discharge period in-depth.

**Methods:**

This work was part of a large, multi-country prospective cohort study, the Childhood Acute Illness and Nutrition (CHAIN) Network. We conducted a qualitative sub-study to understand the post-discharge treatment-seeking and recovery experiences of families of undernourished children aged 2–23 months admitted in a rural and urban icddr,b (International Centre for Diarrhoeal Disease Research, Bangladesh) hospital. Methods included repeat in-depth interviews (73 interviews in total) with 29 family members of 17 purposively selected children. These data were supplemented by interviews with 33 health workers, and by observations in hospitals and homes.

**Results:**

Important drivers of treatment-seeking perceived to support recovery included advice provided to family members while in hospital, media campaigns on hygiene practice, availability of free treatment, and social and financial support from family members, relatives and neighbours. Key perceived challenges included low household incomes, mothers having to juggle multiple responsibilities in addition to caring for the sick child, lack of support (sometimes violence) from the child’s father, and family members’ preference for relatively accessible drug shops, physicians or healers over hospital admission.

**Conclusion:**

Development of interventions that address the challenges that families face is essential to support post-discharge adherence to medical advice and recovery. Potential interventions include strengthening information giving during hospitalization on what post-discharge care is needed and why, reducing direct and indirect costs associated with hospital visits, engaging fathers and other ‘significant others’ in post-discharge advice, and building mobile phone-based support for follow-up care.

## Introduction

Post-hospital discharge mortality is high among children in many low and middle-income countries (LMICs) [1–4], often exceeding in-hospital mortality rates [5, 6]. In Bangladesh, as in other LMICs, studies conducted in icddr,b hospitals have documented post-discharge mortality for children aged under five years at 4.2% and 8.7% for rural and urban areas respectively, with 70% of deaths (rural) and 59% of deaths (urban) occurring within 3 months and 30 days post-discharge respectively [4, 7]. Children with Severe Acute Malnutrition (SAM) and Moderate Acute Malnutrition (MAM) often have co-morbidities such as HIV and pneumonia, and are particularly vulnerable to mortality during hospital admission and after discharge, despite adherence to current WHO (World Health Organization) guidance [1, 3–6, 8, 9]. This has also been observed in Bangladesh [4].

A number of quantitative studies have identified risk factors for post-hospital discharge mortality among young undernourished children in resource poor countries, including Bangladesh, including leaving the hospital against medical advice, poor water, sanitation and hygiene practices, low maternal education levels, breast-feeding practices, non-use by parents of an emergency mobile contact for hospital physicians and care-seeking from traditional healers [1, 2, 4–8, 10, 11]. These quantitative studies were not designed to explore family member and frontline health care provider perspectives on treatment-seeking and recovery post-hospital discharge. Qualitative work can complement quantitative data to support identification of locally appropriate interventions with potential to improve children’s outcomes.

The available literature suggests that treatment-seeking and recovery post-discharge in resource poor settings will depend not only on the child’s physical health at discharge, but also on wider influences across the entire treatment-seeking pathway that shape when, where, and in what condition children are first admitted in hospital, the hospitalization experience itself, and post-hospital discharge actions [2, 4–6, 8, 10].

Important potential influences can be grouped across three inter-related domains: socio-cultural and environmental; economic; and health system or service influences [12]. Socio-cultural and environmental influences include: levels of education of family members; nature of and adherence to local traditions and beliefs; mothers’ decision-making power and access to resources; quality of the household environment including the condition of living areas; and ease of physical accessibility to homes and facilities [2, 8, 10]. Economic influences include: household access to funds to pay for recommended nutritional food for all family members; cost of transport and treatment, including admission and medicine costs; and loss of income associated with caring for children and hospital visits [2, 5, 6, 13]. Health system level influences include: hospital discharging practices, availability of essential drugs in the hospital, quality of post-discharge follow-up, access to emergency transportation facilitating children’s return to hospital, and caregivers’ facility-based experiences [2, 10, 13]. Influences on treatment-seeking and recovery post-discharge often differ between rural and urban settings given their contrasting socio-cultural, environmental, economic, and health system contexts [14–18]. Importantly, many of these influences interact and intersect to present vulnerabilities and challenges for family members in supporting their young children post discharge from hospital. They can also offer opportunities to support and build upon family members’ agency (ability to make choices and to act) and positive outcomes. The ways in which these vulnerabilities, challenges, opportunities and agency come together from the perspective of children’s family members, and frontline providers, is best explored qualitatively.

To our knowledge, no in-depth qualitative study has been carried out in Bangladesh to explore the influences on treatment-seeking and recovery post-discharge from the perspective of undernourished children’s family members and frontline health providers. This study gathered rich accounts of the treatment-seeking journeys that family members followed for their children in two very different Bangladesh sites.

## Materials and methods

### The Bangladesh health system context

Bangladesh is one of the world’s fastest-growing economies and one of the fastest-growing lower middle-income countries [19]. However, according to the World Bank, as of 2016, 14.8% population of the country lived below the international poverty line on less than $1.90 per day [20]. Bangladesh has a highly pluralistic set of health providers across the country, including formal and informal, traditional and modern, and private and public [21]. Within the public sector, the Ministry of Health and Family Welfare operates a dual system of health and family planning service delivery through medical college hospitals, specialized hospitals, district hospitals, Upazila (sub-district) health complexes (with 51 beds), union health, and family welfare centers and community clinics (CCs) [21]. In rural areas, Health Assistants and Family Welfare Assistants assist community health care providers (CHCP) to run CCs, supported by Community Health Workers (CHWs) linked to non-government organizations (NGOs). In urban areas, the ‘Dhaka North City Corporation’ and ‘Dhaka South City Corporation’ (under the Ministry of Local Government) are responsible for providing primary health care services including for the urban poor. Services at this level are offered through a contracted-out mechanism in which NGOs periodically bid to provide low-cost services [22]. In addition, in Dhaka (urban) and Matlab (a rural sub-district), the non-governmental hospitals of icddr,b provide treatment, specializing in diarrhoeal disease [23, 24].

### Study sites

The qualitative study presented in this paper was nested within the Bangladesh site of a large prospective cohort study—“The Childhood Acute Illness and Nutrition (CHAIN) Network”: Building the evidence base for care of acutely ill, undernourished children in limited-resource settings” between March 2017 to December 2018 [25, 26]. The parent study (CHAIN) is a large multi-country observational cohort study conducted in Bangladesh, Pakistan, Kenya, Uganda, Malawi and Bukina Faso, to better understand risk factors and identify potentially modifiable mechanisms to target in interventions to reduce post-discharge mortality of children [25].

The two CHAIN study hospitals in Bangladesh were the icddr,b Dhaka (urban) hospital—located in the capital city and Matlab (rural) hospital—about 55 km southeast of Dhaka, located in a sub-district of Chandpur District [25]. These hospitals provide a full range of free treatment and services for patients admitted with diarrheal disease [27, 28]. Each year over 100,000 diarrheal patients are admitted in Dhaka hospital and between 20,000 and 25000 in Matlab hospital [21–23, 27, 29]. Children with diarrhea and malnutrition are managed according to the hospitals’ management protocol, including the specific guidelines for the management of malnourished (SAM) children [23, 29]. The Dhaka hospital has an intensive care unit (ICU) and a nutrition rehabilitation unit (NRU), with the care given saving an estimated 40,000 lives per year [30]. In Matlab hospital, children with SAM are discharged from the hospital immediately after recovery from their primary illness, or with significant improvement, as there is no NRU available. Critical patients are referred to the Dhaka Hospital or elsewhere as needed.

Most patients admitted to the two icddr,b hospitals are from low socio-economic backgrounds. In Dhaka city, many hospital users live in one of the city’s ‘slums’, where 37.4% of city dwellers live and where environments can be very contaminated [31]. When compared to non-slum urban areas, slum-dwelling children are more malnourished, have lower immunisation rates, and have higher rates of measles and other infectious diseases. They also suffer more often from diarrhoeal illness and severe dehydration, and their mothers are more likely to be out earning an income [32]. The majority of the admitted children in Matlab hospital of icddr,b were from the Matlab sub-district, which is entirely rural, lacks major towns or cities (except for the Matlab bazaar), and has limited inter-village trade and commerce. The dominant occupations of its population are subsistence farming and fishing [33].

### Recruitment of participants and data collection

We adopted a case study approach drawing on interviews and observations focused on family members of 17 children with undernutrition (‘cases’) who had been admitted in one of the two study hospital sites. A total of 488 undernourished children aged less than 23 months were included in the icddr,b CHAIN cohort, 269 in the urban site and 219 in the rural. We (MFU, MAH, MAI) purposively [34] selected 17 ill undernourished children from the main CHAIN cohort participants, ensuring diversity in children by location (8 rural and 9 urban), nutritional status, education of parents and exposure to recent major social disruption such as moving house, death of a parent, and change in the main caregiver.

We conducted repeat in-depth interviews (total interviews = 73) in the homes of 29 caregivers and family members of 17 purposively selected children on days 1–7, 45, 90 and 180 post discharge (Table 1). Repeat interviews facilitated establishment of rapport with participants and supported collection of in-depth information on children’s illness, treatment-seeking trajectories and recovery experiences over that period. It also allowed for some observation of the home environment to supplement data from interviews and understand family context. We (MFU, MAH, MAI) also conducted Key Informant Interviews and FGDs with icddr,b health workers working in the study hospitals and with community health workers (CHWs) working in patients’ communities to gather their perspectives (Table 1). Health workers interviewed had interacted with children’s caregivers and so were able to add to household stories as well as highlight relevant health system challenges. Triangulating our data sources strengthens the trustworthiness of the findings. We collected data until no new topics emerged [35–37]. All interviews were audio-recorded.

**Table 1:** Methodology and participants.

Methods	Participants	Field sites
Repeat in-depth Interviews (73)	Caregivers of children (17)	Dhaka (38)
	Fathers and grandmothers of children (12)	Matlab (35)
Key Informant Interviews (15)	Health workers (icddr,b hospital based)	Dhaka (5)
		Matlab (3)
	Community health workers (CHWs)	Dhaka (4)
		Matlab (3)
Focus Group Discussion (FGD) (2)	Community health workers (CHWs)– 9 participants	Dhaka (1 FGD)
	Community health workers (CHWs)– 9 participants	Matlab (1 FGD)

We drew on treatment-seeking literature summarized in the introduction, and on previous conceptual frameworks [18, 38, 39] to develop a conceptual framework for our study (S1 Fig). The conceptual framework highlights potential economic, health service/system, socio-cultural and environmental influences on vulnerability, agency and treatment-seeking actions and experiences. The interview tools for family members allowed for open narrative of the child’s illness and treatment-seeking journey, followed by probes to cover the conceptual framework topics. The focus of discussion was discharge (timing and decision-making) and post-discharge treatment-seeking behavior and adherence to advice. Health worker interviews were similar, without the child specific open narrative. Initial interviews served as pilots of the tools, which were adapted throughout the study.

### Data analysis

Qualitative interviews were transcribed verbatim, and interview and observation notes written up immediately following fieldwork. We used two complementary approaches to analyze the data: a narrative approach [40, 41] and a thematic coding approach [42], as described in more detail elsewhere [18]. The narrative approach entailed constructing detailed household stories for each child (or case), which offered a rich and in-depth picture of the household itself and the entire treatment-seeking trajectory and experience. Household stories were typed up and translated into English using Microsoft Word. This approach ensured that discharge and post-discharge elements were not extracted and separated from their context and facilitated comparison across households. Second, a thematic approach was applied to the entire data set (specifically a framework analysis approach) where we condensed data from the transcripts through an iterative process of coding, building into categories and themes, with data managed using NVivo11 software. The coding framework was based on the interview tool probes, in turn based on the conceptual framework [S1 Fig], as well as on emerging themes of interest. To support trustworthiness, two researchers coded initial transcripts, compared results, and resolved discrepancies.

### Ethical approval

CHAIN protocols were approved for ethics in all participating countries. In Bangladesh, the study was approved by the Ethical Review Committee of icddr,b. Written informed consent was sought from participants for all in-depth interviews, observations and recordings, and–continuing consent for households–was checked in each subsequent household visit. There were no refusals.

## Results

Following an overview of participant and household characteristics, and of children’s treatment-seeking trajectories and health outcomes, we present the key influences on post-discharge treatment-seeking and recovery from the perspective of children’s family members and frontline health providers. Influences are grouped according to our conceptual framework domains (S1 Fig).

### Participant and household characteristics

The main characteristics of the 17 children are summarized in Table 2. 10 were SAM, 7 were MAM, and most had experienced a disruption in the family, including recent migration, separation or income-earning loss among parents, maternal illness or a change of caregiver. Across both sites, children were admitted into the hospital and enrolled into the main CHAIN cohort aged 4 to 16 months, with all reported by parents as having been sick ‘on and off’ since birth. All children had diarrhea on admission, usually with other symptoms including vomiting (n = 10), fever (n = 7) and pneumonia or severe respiratory distress (n = 5, all urban). Selected children were broadly similar to the wider cohorts in education level of main caregiver, in having no reported income, having low levels of food security and in asset scores. All households reported access to a phone to CHAIN organisers but in our qualitative work we learned that young mothers particularly in rural households were hesitant to use the phone for fear of being accused of extramarital affairs.

**Table 2:** Participants and households’ characteristics (n = 17).

**Urban participants and households**															
**ID**	**Children characteristics**				**Mothers characteristics**					**Households characteristics**					
	**Age, sex, nutritional status**	**Sick since birth?**	**Other illness at admission**	**Caregiver**	**Age**	**# of kids**	**Marital status**	**Education (completed)**	**Employment status**	**Nuclear, extended family, size**	**Social disruption before admission**	**Income source**	**Decision maker**	**Availability of mobile phone, owner**	**Caregiver’s-women access to phone**
HH52	14m, F, SAM	Cough, fever	Diarrhea, fever, pneumonia	Mother	20y	3	Married	Illiterate	Housewife	N, 5mem	Household change	Carpenter	Father	Yes, Father	Yes
HH53	6m, M, SAM	Fever, cough	Diarrhea	Mother	20y	1	Divorced	Primary	None	E, 8mem	Household change	Garment worker	Grandmother	Yes, caregiver, uncle, aunt	Yes
HH55	4m, F, SAM	Fever	Diarrhea, vomiting	Maid servant	19y	1	Married	Primary	Garment worker	N, 4mem	Caregiver changed	Garment worker	Father	Yes, Mother, Father	Yes
HH57	4m, F, SAM	Fever, cough	Diarrhea, cough, fever	Mother	16y	1	Married	Primary	Housewife	E,6mem	Migration from rural to urban, parents sickness	Garment worker	Maternal grandmother	Yes, Father, Grandmother	Yes
HH51	8m, M, MAM	Coldness, diarrhea	Diarrhea, fever, oral thrush	Mother	17y	1	Married	Primary	Housewife	N,3mem	Migration from rural to urban, wage loss	Bakery shop worker	Father	Yes, Father	Yes
HH59	4m, F, MAM	Fever, coldness	Diarrhea, fever	Mother	17y	1	Married	Primary	Housewife	E,5mem	Migration from rural to urban	Garment worker	Father	Yes, Father, Grandfather, Aunt	Yes
HH60	8m, F, SAM	Coldness	Diarrhea, pneumonia	Mother in low	35y	3	Married	Illiterate	Small restaurant business	N,5mem	Mothers’ sickness	Car driver	Father	Yes, Father, Caregiver	Yes
HH62	4m, M, MAM	Diarrhea	Diarrhea, severe respiratory distress	Mother	35y	3	Married	Illiterate	Housewife	N,5mem	Mothers’ sickness, caregiver change	Small business	Father	Yes, Caregiver, sister	Yes
HH63	7m, F, SAM	Breathing difficulties, fever	Diarrhea, pneumonia	Aunt	19y	1	Divorced	Secondary	-	E,4mem	Wage loss	Garment worker	Grandmother	Yes, Caregiver, Father, Grandmother	Yes
**Rural participants and households**															
**ID**	**Children characteristics**				**Mothers characteristics**					**Households characteristics**					
	**Age, sex, nutritional status**	**Sick since birth?**	**Other illness at admission**	**Caregiver**	**Age (years)**	**# of kids**	**Marital status**	**Education level (completed)**	**Employment status**	**Nuclear, extended family, size**	**Social disruption before admission**	**Income source**	**Decision maker**	**Availability of mobile phone, owner**	**Caregiver/women access to phone**
HH04	10m, F, SAM	Diarrhea, fever	Diarrhea, fever	Mother	20y	3	Married	Secondary	Housewife	E, 9mem	Mother’s sickness, twin baby	Day labor	Grandfather	Yes, Grandmother	Yes
HH08	5m, M, SAM	diarrhea, urine infection	Diarrhea, vomiting	Mother	26y	2	Married	Secondary	Housewife	E,10mem	Mother’s sickness	Worker in abroad	Grandfather	Yes. Mother	Yes
HH12	9m, F, SAM	Fever	Diarrhea	Mother	23y	2	Married	Secondary	Housewife	N,5mem	None	Clerk	Father	Yes, Father	Yes
Hh10	14m, F, SAM	Fever, coldness	Diarrhea, fever	Mother	35y	3	Married	Secondary	Housewife	N, 5mem	None	Night guard	Father	Yes, Grandmother	Yes
HH01	11m, M, MAM	Cough & coldness	Diarrhea, fever	Mother	23y	3	Married	Secondary	Housewife	E, 9mem	Mother’s sickness	Worker in urban Dhaka	Grandmother	Yes, Grandmother	Yes
HH02	6m, M, MAM	Fever	Diarrhea, fever	Mother	24y	2	Married	Secondary	Housewife	N, 3mem	Parent sickness, wage loss	Day labor	Father	Yes, Father	Yes
HH03	11m, F, MAM	Fever & urine problem	Diarrhea, vomiting	Mother	25 y	2	Married	Primary	Housewife	N, 4mem	Mother’s sickness	Carpenter	Father	Yes, Mother	Yes
HH05	16m, F, MAM	Coldness, pneumonia & diarrhea	Diarrhea, vomiting	Mother	33y	3	Married	Secondary	Housewife	E, 6mem	Wage loss	Carpenter	Father	Yes, Grandmother	Yes

### Children’s treatment-seeking trajectories and health outcomes: An overview

There had been significant treatment-seeking efforts by most parents by the time of admission in icddr,b hospitals, including visiting drug sellers, healers, private doctors and public hospitals (Table 3). In both rural and urban areas, several treatment-seeking actions had been taken sequentially or in parallel.

**Table 3:** Treatment-seeking patterns for the index child’s illness pre-admission.

	Rural		Urban		Total
	SAM (N = 4)	MAM (N = 4)	SAM (N = 6)	MAM (N = 3)	N = 17
Visited a healer pre-admission	4	4	2	1	11
Visited a drug seller pre-admission	4	4	4	1	13
Visited another facility/doctor pre-admission	2	4	5	2	13
Visited a CHW pre-admission	0	3	0	0	3
Was admitted in another facilities before coming to icddrb hospital	1	2	0	1	4

During initial hospitalization, we observed hospital health workers teaching caregivers about food preparation and preservation processes and complementary feeding. Also covered were maintaining minimum dietary diversity, meal frequency, using a spoon instead of fingers or a bottle for feeding, and avoiding drinking cold food/water. Demonstration sessions were organized on breastfeeding, hand washing, and using treated water for cooking and drinking. On discharge, hospital health workers advised caregivers to continue with prescribed medicine, complete vaccines, breastfeed (noting that employed mothers can store breast milk), and cook homemade food. Caregivers were given health workers’ mobile numbers to contacted them for advice and prescriptions.

13 of the 17 children had fully recovered by the time we completed our interviews; 4 had died (3 urban and 1 rural), one before discharge. Two of the 16 children discharged alive were discharged by parents against medical advice, both of whom were SAM. Post-discharge, most parents reported that children took time to recover, with many experiencing new episodes of illness. Eight visited a healer over the post-discharge period, 14 a drug seller, and nine another health facility. 11 were re-admitted either back to icddr,b or into another hospital (Table 4).

**Table 4:** Discharge and post-discharge treatment-seeking patterns for the index child’s illness.

	Rural		Urban		Total
	SAM (N = 4)	MAM (N = 4)	SAM (N = 6)	MAM (N = 3)	N = 17
Recovered	3	4	4	2	13
Discharged against advice	1	0	1	0	2
Visited a healer post-discharge	2	3	2	1	8
Visited a drug seller post- discharge	3	4	4	3	14
Visited another facility/doctor post discharge	2	0	4	3	9
Visited a CHW post-discharge	0	2	1	0	3
Was re-admitted	1	3	4	3	11

The caregivers of selected children mentioned more treatment-seeking actions and greater use of traditional healers than was observed in the overall cohort, suggesting greater openness and amount of detail in qualitative repeat interviews than a one-off survey.

In subsequent sections, we highlight key influences and impacts on post-discharge treatment-seeking and recovery. However, to illustrate complexities of treatment-seeking patterns and influences, and the interplay of vulnerabilities and abilities, we first present two contrasting household stories: one from an urban and one from a rural household (Boxes 1 and 2). These stories also show how the admission and post-discharge experience for children is inextricably linked to their pre-admission situation and experience.

**Box 1. Story of a rural child aged 14 months with SAM in Bangladesh (HH10).**

Mother had three children and the enrolled child’s birth order is third. Mother age was 35 years, completed secondary education and housewife. Father is a day labor. This is a nuclear family with 5 HH members, living in a one room house they built. Sometimes the child’s aunt provided financial support. They have their own toilet (ring slab) but it looked unhygienic and they collect water from their neighbor’s tube and drinking without treat. Since birth, the child had been suffering from cough, fever and diarrhea frequently and received treatment from healers and nearest drug sellers.

**For this admission,** The child had watery stool for 5 days. The mother presumed, this was because her child drank contaminated water from a bowl left in the courtyard to collect rainwater. When it first began, the father bought medicine the next day from the village doctor, but she did not get well. She got weaker and the frequency of her stool increased from 5–6 times a day to more than double. On the fifth day, the parents decided to take her (child) to icddr,b hospital as per GM and aunt’s suggestion.

- The mother did not report anything difficulty, while in the hospital. The child liked to have milk Semolina that was provided from the hospital. So her mother learned about its preparation process in order to continue to take better care of her child at home. Her diarrheal condition improved within 3 days of admission.
- Before discharge the mother was again counseled about food preparation, feeding practices, child caring, hygiene practices and basic medicine administration procedure. Nurses gave some medicine to continue after discharge.

**After discharge:** The mother prepared food for the child as per hospital’s instruction for about 1.5 months. But it stopped when the father lost his job and they had to depend on savings. The mother’s hygiene practices were also observed to slip over time, and the child was given low quality readymade food because she refused to take the homemade ones.

- The mother did not give her child the medication which was prescribed from the hospital: she felt child no longer required it, since her condition had improved, and that took much medication might adversely affect the child.

After 45 days of discharge, the child was suffering again from fever and cold. The mother thought that it happened because the child frequently played with rain water while there was no one to look after the child. Nevertheless, the mother did not seek any further treatment for the child. She explained “*Since I am also suffering from cold and taking medication for it*, *so my medicine will be transferred to my daughter through breast milk*, *so she does not need to take any further medication”*. The cold persisted till 180 days of discharge from hospital.

**Box 2. Story of an urban child aged 6 months with SAM in Bangladesh (HH53).**

The child was born in a rural area but moved to Dhaka at aged 45 days, 20 years old mother and divorced. They moved into one room with the mother’s maternal family; trying to meet daily needs through garment factory work. At 2 months the child was crying after breast-feeding. The grandmother felt he wasn’t getting enough breast milk and was advised by a nearby drug seller to get formula. But the child continued to deteriorate and by 4 months was skinny and developed measles. To get low cost traditional treatment he was taken back to the rural area, but returned weak and soon developed watery diarrhea. Despite vitamin syrups and later ORS from drug sellers the child’s condition continued to deteriorate and he started to vomit. The drug seller referred the child to a hospital, where he was referred on to icddr,b and admitted.

**For this admission:** The child stayed in the hospital for 20 days. Hospital staff noticed that when the mum was not there, the grandmother often slept, because she herself was unwell. The child was not recovering so the mum and grandmum consulted a diviner about whether or not to leave the hospital, who advised that they stay. Two further major concerns for the mother and grandmother during the admission were who would be cooking for the other household members at home, and the child’s father phoning the mum to say, “*if anything happens to my son*, *I will sue you*”.

**After discharge:** Despite nutrition counseling, the mother felt that breast milk and formula were adequate; that the child did not need complimentary food. The GM admitted she sometimes felt so overwhelmed she wished the child would be sent to his father so her daughter could remarry. Within a week of discharge, the child developed cough and fever, and on day 14 diarrhoea. Relatives and neighbors advised against returning to icddrb in case he died but the mother had faith in them so went. He was re-admitted for 12 days with diarrhea and pneumonia.

The mum prepared food at home as per hospital instruction for 3 months; affordable because the father was issued a court order to provide. But on the 3rd follow-up visit the child appeared sick and thin-his aunt had suddenly lost her job and the mum was suspected to be spending some of the child’s money from the father on herself. His cold at the time was considered by her not to a big problem as he’d had it since birth so he would recover. Unfortunately, the child died not long afterwards.

### Socio-cultural and environmental influences on treatment-seeking and recovery

#### Mothers’ workload in homes and levels of support from others

Many rural and urban women had very heavy household workloads, including cooking and serving meals, washing clothes, rearing domestic animals, and harvesting rice in the paddy fields. Mothers’ need to return to their household work was reported to be an important influence on their decision to discharge their ill children against medical advice. Parents generally felt that since their child’s condition was improving, and was no longer life-threatening, it was a greater priority to get home to their other responsibilities than to stay in the hospital for an additional few days. As one mother explained:

“*While I was staying in the hospital*, *my ability to care for my husband and elderly mother-in-law were disrupted*, *and also my rearing of domestic animals [for income]*. *So*, *I decided I had to take my child home against the doctor’s advice*.*”* Mother, Rural. HH 5.

These workloads also made it challenging for mothers to adhere to nutrition, hygiene, and treatment advice at home and to bring their children back to the hospital or facilities as advised.

Mothers who were able to keep their children in hospital for the recommended period, and to adhere to advice post-discharge, were able to draw on support from other family and community members during hospitalization and post-discharge, with assistance most often coming from husbands, older children, other close relatives, and female neighbors.

Practical support from female relatives and friends was particularly important. For example, four rural mothers visited their own mothers’ home in the immediate post-discharge period to get support from grandparents and aunts in providing medicine, and preparing and providing food as per hospital-physician advice. In one case (HH 08), a maternal grandmother and aunt visited the child’s home to assist the mother with household chores as the mother focused on the child’s care. Some husbands were also reported—and observed—to assist practically:

“*I and my wife have decided to work outside of the home at different times to take care of our child properly*. *So*, *I work at night when my wife can take care of my child and my wife works during the day when I can stay at home to take care of my child*. *I think that both of us need to work to fulfill our basic needs properly and it’s important to look after our child as we love her very much*.*”* Father, Urban, HH55.

In the rural HH12’s case, a close relative who resides nearby a health facility had guided the parents of the child over mobile phone to re-admit their ill child in an urban private hospital in order to get what was perceived as better treatment.

Family and friends were clearly an important source of information and advice for mothers when their children remained sick or did not recover at the desired pace. Some urban mothers were new to the area and therefore unfamiliar with the locality, and so relied particularly heavily on longer term residents’ advice. While some advice was to seek prompt care from trained medical practitioners or from government health care facilities, many mothers reported following advice to purchase medications from drug sellers or general shops or to simply dress their children more warmly. Common purchases for diarrhea were oral saline, and for coughs and fevers were Napa syrup (Paracetamol). Some advice entailed the use of traditional medicines or treatments, as described further below in the beliefs around etiology of illness and related treatment.

#### Abuse of mothers by family members and reported links to depression

Even with support of others, mothers described having to achieve a challenging balance between adequately fulfilling household chores and child care responsibilities with putting aside a specific time and effort to support the discharged child’s recovery. Several expressed concern about the difficulty of maintaining their relationships with their husbands and their reputation in the wider household and community. As one mother explained:

“*Since I have to cook meals three times a day*, *serve food to others*, *[and] clean the households and clothes*, *it is really difficult for me to [also] provide food and medicine*, *breastfeed*, *and bath my child on time*. *After doing household chores all day long*, *I am so tired*, *particularly at night*, *and often feel ill*, *which adversely affects the health care of my child*. *Unfortunately*, *my husband blames my improper childcare for my child’s illness*. *And if I say something to retaliate against my husband’s comments*, *others might treat me as a bad woman or wife*.*”* Mother, Urban. HH52

Several mothers directly attributed their child’s failure to recover quickly to their difficulty in managing all of these tasks. One talked about her child being exposed to rain and cold as a result of her work, and another about hiding her child’s condition from others out of a concern about being blamed for it. Notable was that four different mothers openly reported that their child’s failure to recover resulted from their own exposure to mental and physical abuse from their husbands and mother-in-laws. As one mother described:

“*My husband often beats me unnecessarily when drunk or when I do not provide the amount of money he has demanded from my father’s home*. *So I am really reluctant to provide medicine and food in time [for the discharged child] as per hospital-physician advice [because he will think I have more money I am hiding]*. *I think my husband dislikes me very much and wants our separation*.*”* Mother, Urban, HH5

Three other mothers reported being told by husbands after the child’s discharge not to give medicines at home, or not to leave home to go to health facilities. Such instructions may be based on jealousy of mothers’ potential interactions with other men, or concern that the treatment is an unnecessary cost. Two of these mothers reported having been beaten previously by their husbands for taking their children to medical practitioners despite having been forbidden. They were fearful to do so again. Mothers reported that this abuse, and fear of it, led to them being depressed, and to marital separations, both of which reduced their affection and attention towards the care of their ill children.

#### Beliefs about the etiology of illness and perceptions about the usage of medicines

Some parents were aware of key danger signs and the prevention and treatment of child illnesses, with many discussing having learned about this during their child’s hospitalization through interactions with health workers. Several also mentioned having learned about them through a television documentary broadcast by the Government’s Ministry of health and family welfare.

While many family members made significant efforts to follow the discharge advice given at icddr,b hospitals, it was clear that some felt that the child’s ill-health was caused by a range of alternative causes rather than biomedical or nutritional explanations. Particularly where children were felt not to be recovering, some parents selected additional or alternative treatments. For example, there were descriptions of children’s failure to recover or a new illness being caused by a supernatural air having a spiritual power over the child, or by a child being affected by an ‘evil eye’ cast by someone in the community (including via the mother through breastmilk). A mothers’ spicy diet was also felt by some to result in diarrhea in their breastfeeding child.

“*My child suffered from diarrhea because I ate spicy food which was transmitted to the body of my child through breastfeeding*. *So I discontinued breastfeeding my child*.*”* Mother, Rural. HH07

Beliefs about what caused the child’s ill health, together with advice of family, friends and neighbors, influenced treatment-seeking decision-making and action. For example, a child believed to have diarrhea caused by an evil eye was taken to a spiritual healer who provided a blessed metal object to tie around the neck and hands of the children or mothers. Children believed to have diarrhea caused by supernatural air were given herbal medicines and sacred water to drink. As with the above example, some mothers opted to discontinue breastfeeding and switched to formula milk.

#### Households’ environment: Water, sanitation and hygiene practices

Children’s family members and community representatives felt that household and community environments undermined children’s recovery, particularly in the densely populated urban slums where there is poor waste management, drainage and ventilation.

“*Please let’s see in the slum*: *a discharged child is walking and playing with the dirty logged water and sometimes putting water into her mouth*. *In this area*, *we often have water trapped after rain because of the inadequate drainage systems*. *And the water is filthy*, *contaminated by dirty things such as open sewers*, *urine*, *and home debris*. *Sometimes we even see open stool in the water*. *Can you imagine how dangerous that is for children*?.*”* Community health worker, Urban, KII-51

In these very challenging social and physical environments, some caregivers were observed to have less-than optimal hygiene practices that may have contributed to the spread of diseases; for example using toilets while barefoot, and washing hands without using soap before going on to feed children:

“*The mother left the child with an older kid and went to the toilet barefoot*. *Having used it she went to the tap and … washed her hands and legs using only water [no soap]*. *Then she went to the kitchen for cooking and after 20 minutes came back to the living room and started feeding food to her child*.*”* Household observation, Urban, HH55

### Economic influences on treatment-seeking and recovery

In the majority of the households, where money was needed or costs were incurred across the treatment-seeking pathway, the father of the child was generally considered to be responsible for meeting such costs. Men played a more prominent role in supporting mothers financially than assisting with other forms of support. Where the sick child’s father was absent, or was unable or did not want to assist, the range of relatives highlighted in the previous section were important. As one mother mentioned:

“*My father sends money (2500 BDT) to me for treatment for my child*, *as he knows well about the financial inability of child’s father for treating the child*.*”* Mother, Rural, HH5

Mothers in particular had very little access to money in homes, and where they earned an income this was primarily through casual and informal work. Many families were largely living ‘hand to mouth’, juggling multiple demands on their cash, with few savings to draw upon. This influenced their choice of action:

“*I have to find money for accommodation*, *food and clothes from my limited [regular] income*. *A sudden treatment and transportation cost to bring the child to the hospital is an extra burden for me*. *The situation is the worst fifteen days into a month when my payment has been used up and when I’m under pressure to repay loans*. *[so with the child’s illness] we went for low-cost treatment from the nearest drug shop*.*”* Mother, Urban. HH53

In contrast with private facilities which are relatively expensive, the free treatment offered at icddr,b—together with the institution’s services and reputation for quality care–were reported by many family members as a major incentive to seek care there when it was needed post-discharge. Despite this, mothers still faced affordability challenges as a result of transport costs to the facility and the need to purchase basic necessities not required when staying at home—mentioned by CHWs and family member. Concerns about these direct and indirect costs, layered on top of the previous treatment-seeking action costs, contributed to some parents discharging their children against medical advice (particularly in the urban area), and to parents being hesitant to bring their children back to hospital post-discharge. As one mother mentioned:

“*I did not communicate with the hospital physician of icddr*,*b using the mobile phone number I was given because I thought they may request us to go there and be re-admitted*. *We did not have enough cash to pay for travel to the hospital and to cover other costs needed during admission*. *The costs we’d already had to meet before the admission [initial hospitalization] were the reasons we were short of cash already and I could not manage to find the money we would need from neighbors and local NGOs because we had recently moved from the rural area [so didn’t have anybody to approach]*.*”* Mother, Urban, HH62

Nonetheless, some families were able to navigate around these challenges of indirect costs by communicating among themselves and with health workers via phone as illustrated in the following quote:

“*I explained clearly over the mobile phone to the child’s father about the loose stool*. *He brought oral saline straight after consulting with a doctor*. *I think this is cost-effective for us as we did not have to pay any transport costs to see the doctor and it was a time saving solution also*.*”* Mother, Urban, HH59

Health workers and fathers of the children mentioned—public hospitals, although officially free, were reported by some to require informal payments, such as a small financial incentive to ensure children are seen during ward rounds. Also, physicians in some public hospitals were reported to encourage unnecessary but relatively expensive diagnostic tests or medicines outside the hospital, and there were some reports of brokers being employed by public doctors to attract families to private facilities. As a rural community health worker explained:

“*Some physicians of public hospitals nominate people [such as village doctors*, *rickshaw puller/transport drivers*, *or hospital health workers] to motivate the parents of the children on the way to the hospital and even during re-admission to bring the patient to the physician’s private facility*. *Sometimes they [public hospital physicians] prescribe unnecessary diagnostic tests and poor quality medicines because they are incentivized to do so by the diagnostic center and drug company*. *[Some parents are aware of these practices] and so are reluctant to seek treatment from public hospitals*.*”* Community representative, Rural. KII68

Families’ treatment-seeking stories suggest that a major reason for treating children at home, or for seeking treatment from untrained medical practitioners (such as drug sellers and healers)—even when recognizing that the care might be sub-standard—is a concern about anticipated costs from hospitals.

Mothers’ need to return to their income-earning tasks and to make up for income loss during admission contributed to their need for support from others to care for their child post-hospital discharge.

### Health system influences on treatment-seeking and recovery

Costs related to the health system and their influence on treatment-seeking and recovery have been alluded to in the previous section. Also important at the health system level are the perceived quality of care at facilities, and–although raised more by health workers than family members—the reportedly heavy use of antibiotics in many households and communities.

Regarding the perceived quality of care, in general, family members described being very happy with their children’s care in icddr,b hospital. In particular, they appreciated being asked directly about the child’s illness history from health workers, and the supportive and respectful interaction with them in the counseling and demonstration sessions for breastfeeding, complementary feeding and hygiene practices. Many family members mentioned that these sessions improved their health care knowledge and encouraged them to adhere to the treatment advice, and that their new knowledge supported their children’s recovery. For example, three mothers felt that their children’s diarrhea cases had eased off because they now wash their hands with soap, and have switched from bottle feeding to feeding with a clean spoon.

This was in contrast to reports of care received in public hospitals. For example, mothers in three homes who had children re-admitted to a public hospital reported being upset that physicians prescribed medicines based only on talking to the duty nurses rather than to the mothers themselves; as mothers they felt they had a far better understanding of the child’s history than the nurses. Such observations and concerns among mothers undermined their trust in the quality of care being offered, and contributed to their discharging their own children against medical advice and taking them either home or for treatment elsewhere. As one father explained:

“*Physicians of the public hospital in Dhaka city are not serious enough to provide good quality care through enough attention and proper interaction with us*. *This happens because the physicians think that it’s usually only poor patients that come to this hospital*, *and their families do not have any political power*. *Besides*, *the physicians in Dhaka city are really powerful themselves*. *So they know nothing will happen [to them]*, *even if a child dies because of their improper treatment*.*”* Father, Urban, HH57

Over-use of antibiotics was regularly raised by health care workers as an influence of recovery, and as resulting from treatment-seeking actions and advice to family members from friends, neighbours and improperly trained or motivated health providers. Hospital health workers were concerned about treatment failure among some children during re-admission due to the unnecessarily high use of antibiotics prior to admission. Many private physicians and drug sellers were perceived to provide these too early in an effort to build their reputation (and profit) in the community through children getting better fast, and to switch the type of antibiotic far too often. Community medical practitioners of public health care facilities felt that they could not compete with these fast-acting drugs given their recommendation of relatively long treatment courses. Another related challenge is that even where the physicians do not prescribe antibiotics, many family members buy them over-the-counter anyway as they are unaware of the potential individual and public health harms of overuse.

## Discussion

As part of a larger inter-disciplinary study, we conducted a qualitative social science study to understand the admission and post-discharge treatment-seeking and recovery experiences of families of undernourished children aged 2–23 months admitted in two icddr,b hospitals in Bangladesh. We gathered perspectives of family members of admitted children to complement past and on-going quantitative data on risk factors for mortality post-discharge. We used a conceptual framework (S1 Fig) to explore family member and provider perspectives of socio-cultural and environmental, economic and health system influences related to opportunities and challenges of post-discharge recovery and treatment-seeking behavior.

Overall, we identified several important opportunities related to treatment-seeking and recovery from the perspective of family members of admitted children. These include: positive and supportive interactions between health workers and caregivers (including valued advice being given through face-to-face information and sessions and phone consultations); free treatment at the study hospitals; and financial and practical support to mothers from family members, other relatives and neighbours. Challenges for treatment-seeking and recovery were mothers having to juggle multiple domestic responsibilities in addition to caring for the child, difficulties in accessing funds, and the lack of support from the child’s father and others. In some cases the latter was related to mothers being concerned about being stigmatized for being a ‘bad mother’ or ‘bad wife’, or physical violence. These challenges were often in the context of unhealthy physical environments and in homes without easy access back to care. We discuss three cross-cutting issues in more detail in this paper: early discharge from hospital against medical advice; post-discharge treatment-seeking behavior and recovery; and concerns about inappropriate use of antibiotics. In a separate paper [43], we explore the gender issues and influences in more depth, highlighting a complex web of gender related influences at health systems/services and household/community levels with important implications for young children’s recovery post-discharge. At household/community levels, include gender roles in homes (with women typically primary caretakers for children, and male family members having a dominant decision-making role in relation to food and treatment-seeking), and an indication of greater reluctance among parents to invest money and time in the treatment of female children, as compared to male children. Further contextually relevant gender analyses are needed to improve health-related interventions, programs and policies [43, 44]. In another paper (submitted) [45], we explore the potential role of community health workers (CHWs) and primary health care centers to support caregivers in supporting post-discharge recovery of undernourished children, and in particular the potential to strengthen the role of CHWs.

Early discharge from hospital by a parent against medical advice is significantly associated in Bangladesh as in other developing countries with higher post-discharge mortality and re-admission [4, 6, 46]. In line with a previous study conducted at icddr,b hospitals [4], we found that mothers who were taking their children home against medical advice were doing so for a range of inter-related reasons, including a view that the child had either recovered enough to go home (ie the child’s condition was improving or he/she had recovered from the primary illness), or was not going to recover and so should be taken home. Further reasons identified through this study included mothers’ or other family members’ concerns about other children and chores at home, worries about indirect costs of admission and–especially in the urban setting—loss of income during admission. Mothers who were able to keep their children in hospital until formally discharged were following advice given during the admission, often with financial and practical support from family members, female neighbors and close relatives. This group was energized by observing their child’s continued recovery. These findings suggest that early discharge is difficult to prevent through even a combination of facility-based interventions. Nevertheless, careful information and communication throughout the admission on the child’s condition, anticipated length of stay and treatment-needs appears essential; as does supporting mothers to communicate family members at home (for example by phone). In addition, in the study hospitals, conditional cash transfers (CCT) targeted at the most vulnerable children or households may help to reduce early discharge by addressing some of the indirect and opportunity costs (such as costs to do with for example transport costs and loss of income respectively) [18, 45, 47]. Nevertheless, we have significant concerns about the sustainability and cost-effectiveness of such programmes, even where they are shown in trials to have a positive impact [47, 48]. Thus, any such intervention would need to be very carefully targeted, with careful tracking of potential unintended consequences.

In some settings, discharge of children against medical advice has been reported to result from inadequate beds, conflict between parents and staff, lack of medicines or food in hospitals, and from high admission costs [2, 10, 13]. Caregivers voiced concerns such as these in relation to public hospitals in Bangladesh (where parents reported that they had discharged their children early because of concerns about informal charging and poor quality of care), but we did not hear about this in the study hospitals, where family members’ descriptions of care were much more positive. These findings need checking with a specifically designed study. However, they suggest that important potential interventions in public hospitals may include initiatives that reduce any unnecessary referral to relatively expensive private facilities and that reduce ‘informal payments’ (i.e. contributions that are additional to those stipulated in institutional policies). Initiatives that increase the presence of health workers in hospitals and that improve health worker-parent communication may also be needed.

With regards to treatment-seeking and recovery post-discharge, adherence to advice given in hospitals is vital. Past studies at icddr,b hospitals in Bangladesh have found that failure to follow advice was associated with mortality of children post-discharge; specifically care-seeking from traditional healers, and failing to contact the hospital physician by mobile phone in an emergency as advised [4]. Many mothers valued the advice they were given during the admission period and on discharge, and were able to follow it post-discharge. However, we noted several other reasons that families fail to follow advice, even where that advice appears to be given well and instructions are demonstrated and appreciated. Important immediate influences included mothers returning to busy workloads in homes, and—particularly in urban areas–mothers needing to earn an income. These responsibilities made it challenging for mothers to offer childcare as advised, and often led to mothers having to hand over care to others in households who may not be as well informed or motivated as mothers to adhere to advice. These caregivers, together with (often male) decision-makers in homes, are influential in what care children receive, and when and where follow-up care is sought, making it challenging for mothers to exert their demands. These findings highlight the importance wherever possible of including significant others–in particular children’s fathers and grandfathers—in health education messaging targeting the post-discharge period. As we have described elsewhere [38], engaging fathers can be valuable in building their trust in the follow-up and advice post-discharge, and their support of mothers. Although we did not examine advice and adherence at public hospitals, broader comments in interviews on poor interpersonal communication suggest this might be an issue. This needs to be specifically examined, given that these facilities serve the majority of the Bangladesh population.

We found that additional influences on adherence to advice post-discharge, as also noted in other studies [2, 4], were physical and financial access to health facilities. Elsewhere, we have highlighted that this is particularly an issue where there is a need to recoup initial admission costs, and where household decision-makers are reluctant to allow their wives to have interactions with male health providers [18, 43]. Being able to seek emergency advice by phone potentially eases such challenges. The families we talked to post-hospital discharge highly valued having the option to receive emergency advice by mobile phone. Although one family opted against this advice because of concerns about opportunity costs, for some families the ability to discuss the child’s situation contributed to significant cost-savings. There is currently widespread interest in Bangladesh and elsewhere in offering advice and support by mobile phones to strengthen adherence to advice in resource-poor settings [4, 49–55]. A recent pilot study in Bangladesh has suggested that mobile phones are a feasible and acceptable option for changing perceptions on nutrition among low-income rural families [55]. We support others [4] that a health worker or community health worker maintaining mobile contact with families of vulnerable children has the potential to support post-discharge adherence to treatment advice, as part of a wider set of interventions. As explored in more detail in a separate paper [45], CHWs could provide a communication link for families between hospital staff and primary health care facilities, including through being reachable by phone, and being able to make phone contact themselves with facility staff.

Inappropriate use of antibiotics by family members and untrained health providers were regularly raised as a concern by health workers in our study, but less so by family members. As has been documented in other settings [56], family members were generally keen to access antibiotics for early recovery of their children, appreciating their convenience, perceived effectiveness and associated savings. Overuse of antibiotics is a major concern globally, contributing to antimicrobial resistance and to increasing costs for families, health systems and for public health more generally [57]. Bangladesh has an estimated 100 000 licensed retail drug shops and a further 100 000 unlicensed drug shops selling prescription-only drugs including antibiotics [58]. Polypharmacy, or the prescribing of 3 or more drugs, is common across urban and rural health facilities (46% and 33% of users respectively) [59]. Providers are responding as elsewhere to customer demand, habits and profit [60], as well as to pharmaceutical companies’ strategies of assigning medical representatives to motivate physicians through inducements, persuasion, and emotional blackmail to prescribe antibiotics so that they can achieve individual and company targets [58, 61]. We agree with others [58, 59] that it is crucial to sensitize parents, medical practitioners and drug sellers regarding the use, prescription and selling of antibiotics particularly for infants and young undernourished children. Given the rest of our findings, such initiatives must be complemented by other interventions that tackle the multiple socio-cultural and environmental, economic, and health system factors that interact to influence family members to choose this relative cheap and easily accessible form of treatment.

### Strengths and limitations of the study

We collected data using multiple qualitative data-collection techniques including individual interviews, observations, and group discussions in two research hospitals, communities, and households of children who had been admitted. Although we only interviewed family members of 17 children, repeated interviews in homes helped us build trust with family members and access rich accounts of the treatment-seeking journeys they had followed for their children. Using multiple methods helped us to understand different perspectives and triangulate findings, and through including an urban and rural site, we were able to see some similarities and differences across very different settings in Bangladesh. Future publications will merge quantitative and qualitative CHAIN data, and include cross-country comparative work.

Although, we did not gather in-depth data from public hospitals, we often heard about experiences in public hospitals. These discussions suggested that many of our findings are highly applicable to those hospitals. However, a specifically designed study is necessary to explore this in depth, and identify potential interventions for public hospitals.

## Conclusion

Children’s caregivers, usually mothers, navigate diverse socio-cultural and environmental, economic and health system challenges in their post-discharge treatment-seeking for their children. These challenges intersect to present complex layered vulnerability to early discharge of children from hospital against medical advice, to non-adherence to follow-up advice, and to provider concerns about an inappropriately high use of antibiotics. Particularly important influences on treatment-seeking and recovery were levels and types of advice and support given by health workers and family members (in hospitals and in homes respectively), access to funds, and costs of care.

Many treatment-seeking actions indicate parents’ efforts and agency that might be built upon to improve outcomes. However, this agency is constrained by difficult family situations and broader structural drivers such as low access to income and gender roles and relations. Interventions with potential to build on parents’ agency and reduce children’s vulnerability to poor outcomes include: strengthening information giving during hospitalization on what post-discharge care is needed and why, and on the risks associated with delays in follow ups and mis-use of antibiotics; initiatives to reduce direct and indirect costs associated with (re)visiting hospitals (including carefully designed cash transfer initiatives); engaging fathers and other influential family-members in discussions on post-discharge advice; and building mobile phone-based support for follow-up care. Given intersecting influences and vulnerabilities, interventions must be integrated.

## Supporting information

S1 Fig Conceptual framework on key influences on post-hospital discharge treatment-seeking and recovery. (DOCX)

S1 File Topic guide for interview with family member. (DOCX)

S2 File Topic guide for interview with health worker. (DOCX)

S3 File Topic guide for interview with CHW. (DOCX)
