# Supplementary material for: Treatment-seeking and recovery among young undernourished children post-hospital discharge in Bangladesh: A qualitative study
Source: PLoS One. 2022 Sep 23;17(9):e0274996. doi: 10.1371/journal.pone.0274996 (PMC9506605; doi:10.1371/journal.pone.0274996)
Supplement: S1 Fig — (DOCX) [file pone.0274996.s001.docx]

**Treatment-seeking & recovery**

Discharged as per hospital protocol

Vulnerabilities and agency

Vulnerabilities and agency

Adherence to advice post discharge

**Affordability of health care**

- Household resources
- **Direct costs** (e.g. treatment cost, transport cost)
- **Indirect costs** (e.g. wage loss, opportunistic cost)

**Health services/system issues**

- Location & available treatment facilities
- Service pattern
- Treatment advice & referral system
- Interaction between patient & provider

**Socio-cultural and environmental influences**

- Tradition/law/customs
- Gender and other social relations
- Household environment
- Childcare practices

**Fig 1. Conceptual framework on key influences on post-hospital discharge treatment-seeking and recovery**
