# Supplementary material for: Treatment-seeking and recovery among young undernourished children post-hospital discharge in Bangladesh: A qualitative study
Source: PLoS One. 2022 Sep 23;17(9):e0274996. doi: 10.1371/journal.pone.0274996 (PMC9506605; doi:10.1371/journal.pone.0274996)
Supplement: S1 File — (DOCX) [file pone.0274996.s002.docx]

**Topic guide for Household Interviews**

**A - Can I begin with some general questions about you, your household and your child**

- *Can you tell me a bit about yourself? eg*
  - How long have you lived in this house? Where were you before then? For what reason did you move here? If married, does the husband also live here or does he live elsewhere?
  - How does the homestead overall function – do you all eat together? Or are you several different households living together?
  - How many children are you currently primarily responsible for? How old are they? Are they living here?
  - Do you normally go to work somewhere during the day? Eg business?
  - While you have to be out of the home who normally looks after your children? How does that set up work for you?
- *To talk specifically about child x XX; i.e. child admitted recently and part of CHAIN) (mapping) - Who’s currently normally involved in caring for child x?*
  - physically feeding him/her
  - looking after him/her during the day
  - providing an income for him/her
  - taking him/her to health facilities if needed

**B - Child Xs health: I’d like to hear the story about your child and his or her health in as much detail as you can remember, right from the point you first began to think that there was something wrong up to where you are today …**

(AND/OR … *How has your child’s health been since birth? OR You landed up taking your child to hospital recently…, can you tell me all about what happened until you felt the need to take him or her there?)*

- - How is your child now? What makes you say that?
  - When did it first become clear that something was wrong?
    - Who first noticed something? What did you think was happening?
    - What did you decide to do and what made you decide to do that? Was there anyone else involved in making that decision?
    - What happened next? What happened before then? What else did you do? (What other actions did you take?)
  - How well did your actions work? (What happened after you took that initial action?)

**For interviewer: If helpful, imagine or even draw the timeline points of the story to facilitate probing**

| ***BIRTH*** | ***Eg healer*** | ***Eg prayers*** | ***Eg private*** | ***Eg duka*** | ***Eg health centre*** | ***HOSP*** | ***INTERVIEW*** *(at 0-7 days post-discharge)* | ***INTERVIEW***  *at day 45* | ***INTERVIEW***  *at 90 days* | ***INTERVIEW***  *at 90 days* |
| --- | --- | --- | --- | --- | --- | --- | --- | --- | --- | --- |

***For interviewer:*** Where possible, look for opportunities to ask things like ‘was that different what you noticed from others of a similar age in the household and community?’ ‘What do you think contributed to those differences’?

**C - Focusing on/asking more about the recent hospitalisation of child x**

- What made you decide to take your child to the hospital at that point? Eg Did you consult with others (who and why?) Eg was it something about how your child looked or behaved?
- Can you tell me about what happened at the hospital, from the point you got there to when you were discharged?
  - Eg what happened when you first arrived? Who from the facility handled your child? What do they do and say?
  - When you left home and were going to the hospital were you expecting that the child would be admitted? What makes you say that?
  - What happened over the course of the admission? How long were you there? What was done with/for the child? And what about with/for you? Eg treatment, advice, support from health workers?
  - Did friends/relatives help you out? At the hospital or at home? If so, how? (eg anyone with your other kids at home? Anyone visiting you with food, money, things).
  - What happened at discharge?

Overall, over the course of the admission:

- - **Overall, what did you think** about how your child was looked after by staff at that visit?
  - Were there any **good thing**s/ways that you or your child benefitted from that visit? What were these?
  - Were there any **difficult things** that were unpleasant, or strange, or that made you worry or feel upset? If so, what were these? Did anybody help you deal with these worries/concerns? (eg staff, friends/family)
  - Were you given any information on what the problem is and what had caused it? Did they have any ideas for you about how to improve the child’s situation? What did they say? Did it make sense? Was it achievable?

**D - Going back in time through the ‘health pathway’**

| For **each previous treatment action** (e.g. herbalist/ divine intervention/ clinic / hospital ) | - What made you **decide on that action**? Did you consult with others and why? - Can you tell me about **what happened** at this visit? - Were you given any advice on what the problem is and what had caused it? Did they have any ideas for you about how to improve the child’s situation? - Were there any **good thing**s/ways you benefitted from that visit? What were these? - Were there any **difficult things** that were unpleasant, or strange, or that made you worry or feel upset? If so, what did you about this? Did family members also do anything about these worries/concerns? How do you feel about these now? - **Overall, what did you think** about how your child was looked after? |
| --- | --- |
| *Further probes (positive & negative responses)* | - Emotional reactions, including fears/embarrassment about disclosure/stigma, positive feelings from support from others at clinic (staff and peers)   - Economic & financial costs, including time spent travelling, and what was missed (including schooling or work)   - Institutional factors underlying the above, including attitudes of staff and styles of communication. |

**E- Back to general about the index child to complete…**

- Overall, what has **worried you most** over the course of your child’s illness, and what has most **relieved you or eased your worries** (and how)?
  - What impact has the child’s health situation had on 1) the main carer(s), 2) the other children, and 3) the wider family
  - To what extent have these worries related to your child having been ill, or would have been there anyway/other reasons?

Possible interventions

- ***As a parent/carer,*** what do you think would make a good positive difference for children who have/had a similar illness to your child?
  - In terms of what might be done in hospitals and other health facilities, in communities, and in homes?
