# Supplementary material for: Treatment-seeking and recovery among young undernourished children post-hospital discharge in Bangladesh: A qualitative study
Source: PLoS One. 2022 Sep 23;17(9):e0274996. doi: 10.1371/journal.pone.0274996 (PMC9506605; doi:10.1371/journal.pone.0274996)
Supplement: S2 File — (DOCX) [file pone.0274996.s003.docx]

**Topic guide for interview with Health Worker (facility based)**

| ***Background information*** | - Can include: Age, gender, level of education, professional qualification, years in the health sector; current role & responsibilities |
| --- | --- |
| ***Perception of HW regarding children admitted with malnutrition*** | - What kinds of children tend to be admitted to facility X with malnutrition? - Is there a typical type(s) of child/family? - Why this particular kinds of children? |
| ***Influences (Positive or Negative)*** | - What are some of the main influences on children being admitted or re-admitted to facility X with malnutrition? (Timeliness of seeking care?) - What are the main influences on recovering or persistently failing to thrive post-discharge? And why? |
| ***For each type of influence – that improves and that fails or even adds to problems***  ***Health facilities, households and communities*** | - What happens? Tell us more. Why does that happen and how does that influence a child’s trajectory? - What do you think is positive that should be built on more? How might that be done? What would be the challenges? Who would be supportive/not? - What is not working well/ should be improved? How? What opportunities/challenges? |
| ***Action taken by, and experiences of, the HW*** | - What did they actually? What care actually provide? Any advice? And why? (The kinds of treatment offered) - How they felt about the experience? |
| ***Health system probes*** | - The kinds of treatment offered - How long people have to wait for care/treatment - The way they are talked to and the information they are given   - Able to understand it?   - Able to act on it? Why/why not? - Whether or not they have relatives present - Costs and affordability - Follow-up/ referral post-discharge (e.g. whether referred in the first place, and whether encouraged to return etc.) - HW’s perceptions of re-admitted patients - What are some of the challenges faced by these families at the facility level? And how do they handle the mentioned challenges? |
| ***Recommendation (move to last row)*** | - What is working well/ should be improved? How? What opportunities/? (Both at health facility and HH level) |
| ***Household/community probes (learned through interaction with the caregivers)*** | - Parents’ understanding of the child’s problem and their interest in seeking biomedical care? - Levels of assistance and support within households – e.g. husbands and wives; different wives (polygamy); other relatives - Support and assistance beyond households? - Poverty levels? Crop failures/issues? - What are some of the challenges faced by these families? - Why does it only affect some and not all households? - Why only some children within households (if that is indeed the case?). - How do families handle challenges faced in the households? |
| - ***Discuss challenges identified by and presented to health workers, and their perceived responsibilities in such circumstances.*** | - As health or research staff, do you hear about or get told about children’s social and economic situations? - What kinds of situations and how do you get to know about them? Please give us examples of common situations that you come across? Are there different issues related to social issues? Economic issues? Facility interactions/quality of care? |
| ***HW response towards identified circumstances*** | - For the most common/pressing example(s) under each:   - How do you respond to these kinds of issues, if at all? Why/why not? Should/can you do anything (more)?   - Are there any implications of these actions? What do you do to address them?   - How might things be done better in the hospital to better support children and their families?   - Who should introduce such changes and how? |
|  | - What about **uncommon social/economic situations that** you have encountered over the last year or so that have really worried you (beyond what you’ve already told me)?   - Probe as above - Remember to explicitly ask about recommendation |

Back to general to complete…

- ***Areas of potential intervention***
  - ***Overall, what do you think needs to be changed or what do you think would make the biggest positive difference for children with malnutrition***? I.e.
    - What might be done in hospitals and other health facilities, in communities, and in homes?
    - What about in terms of interactions with researchers?
