# Supplementary material for: Treatment-seeking and recovery among young undernourished children post-hospital discharge in Bangladesh: A qualitative study
Source: PLoS One. 2022 Sep 23;17(9):e0274996. doi: 10.1371/journal.pone.0274996 (PMC9506605; doi:10.1371/journal.pone.0274996)
Supplement: S3 File — (DOCX) [file pone.0274996.s004.docx]

## Topic guide for interview/FGD with Community Health Worker (CHW)

| Turn on the voice recorder if the participant is okay with it (if participants gave consent to use it), or take details note by note taker during interview.  **A - Discharge and referral policies for undernutrition (any knowledge or roles)**   - For the children managed in-patient, do you know what happens **at discharge** in terms of information given and referral? - Do you know whether that is what is supposed to happen (policy) or people just do it? - Do CHWs play a role in any of these processes at the hospital? What are these roles?   - Any contact with MAMs/SAMs?– is this your responsibility or just what you do?   - How does it (specify...what) work? For referral, is the link direct with homes or through peripheral facilities or CHWs?   - How important do you feel it (specify...what) is? Do other types of volunteers or staff have this role?     - What works well? What are challenges?     - Eg appreciated by families?   **B – Other hospital based roles and post-discharge responsibilities if any**   - Any other involvement in hospital activities   - – eg discharge for patients?   - What about your other activities in the hospital (icddr,b and other hospitals and nutritional recovery units) whether disease focused, receiving patients and referrals, communication with other facilities about the patients.   **C. After discharge:**   - Do you get to hear about the children after they have been discharged from the hospital? How? And what happens then? - How do you get to hear of them (eg written, phone call from hospital or patients mother)?   - Is this a common practice?   - If not, what is supposed to happen? What usually happens? Why? Tell me in detail. - What do you do then?   - Do parents appreciate this? Why or why not? - Do you get to follow-up any other children up after they have been discharged from the hospital? How? - Is this generally your work/responsibility? If not how much of an additional workload does/would this constitute? - What are the factors that influence CHWs involvement in following up children after discharge?   - Positive(facilitators)   - Negative (barriers) - Do CHW personal characteristics have a role to play in this eg age, gender and how? what about the health systems factors eg integration, payments/incentives, workload, training, supervision?   **D – To create/improve the formal role of CHWs in supporting children with undernutrition on and post discharge**   - How would it function? - What would be needed by CHWs from facilities to support CHWs? From families?   - Would CHWs personal characteristics have a role to play in such a programme? eg age, gender and how?   - Overall challenges envisioned with working with malnourished children post hospital discharge including support, resources and workload   - Use of mobile phones: what role does/can mobile phone technologies play   - What specific problems would emerge with working with these particularly vulnerable populations (ethical dilemmas). - **Stakeholders:** Who would be involved in such a programme or who supports your work? How? |
| --- |
